# Expression Analysis of NF-κB-Related lncRNAs in Parkinson’s Disease

**DOI:** 10.3389/fimmu.2021.755246

**Published:** 2021-10-13

**Authors:** Soudeh Ghafouri-Fard, Mahdi Gholipour, Atefe Abak, Mehrdokht Mazdeh, Mohammad Taheri, Arezou Sayad

**Affiliations:** ^1^ Department of Medical Genetics, School of Medicine, Shahid Beheshti University of Medical Sciences, Tehran, Iran; ^2^ Phytochemistry Research Center, Shahid Beheshti University of Medical Sciences, Tehran, Iran; ^3^ Men’s Health and Reproductive Health Research Center, Shahid Beheshti University of Medical Sciences, Tehran, Iran; ^4^ Neurophysiology Research Center, Hamadan University of Medical Sciences, Hamadan, Iran; ^5^ Skull Base Research Center, Loghman Hakim Hospital, Shahid Beheshti University of Medical Sciences, Tehran, Iran; ^6^ Urology and Nephrology Research Center, Shahid Beheshti University of Medical Sciences, Tehran, Iran

**Keywords:** Parkinson’s disease, lncRNA, NF-κB, expression, biomarker

## Abstract

Parkinson’s disease (PD) has been shown to affect approximately 1% of the persons aged more than 65 years. This multifactorial disorder has been associated with abnormal function of NF-κB signals. In this research, we have evaluated expressions of NF-κB-related long non-coding RNAs in the circulation of PD patients compared with healthy controls. Expression of *PACER* was lower in total PD patients compared with healthy persons (Ratio of mean expressions (RME)=0.32, P value<0.001). This pattern was also evident among males (RME=0.25, P value<0.001). Expression of *DILC* was higher in total PD patients (RME=4.07, P value<0.001), and in both sex-based subgroups (RME=3.77, P value=0.01 and RME=4.25, P value<0.001, for females and males, respectively). Similarly, *CEBPA* was significantly over-expressed in total PD patients (RME=14.76, P value<0.001), and in both sex-based subgroups (RME=12.42, P value<0.001 and RME=15.80, P value<0.001, for females and males, respectively). ATG5 had a similar expression pattern (RME=2.6, P value=1E-08, RME=1.73, P value=0.03 and RME=3.09, P value=1E-07, for total cases, females and males, respectively). H19 was up-regulated in total cases and male cases compared with corresponding controls (RME=2.19, P value<0.001, RME=2.68, P value=0.01, respectively). Finally, *HNFA1-AS* was down-regulated in all comparisons (RME=0.10, P value=2E-06, RME=0.08, P value<0.001 and RME=0.12, P value<0.001, for total cases, females and males, respectively). Among PD patients, expressions of *NKILA* and *ADINR* were robustly correlated with each other (r=0.75, P value=2.40E-10). In addition, expression levels of *DICER1-AS* were significantly correlated with those of *ADINR*, *PACER* and *H19* in these patients (r=0.73, P value=1.76E-9; r=0.72, P value=5.15E-09 and r=0.72, P value=3.09E-09, respectively). Correlation analyses among healthy controls revealed robust correlations between *CHAST* and *CEBPA* (r=0.84, P value=3.09E-09), *NKILA* and *ADINR* (r=0.80, P value=4.24E-12) as well as between *DILC* and *CHAST* (r=0.76, P value=1.70E-10). *CEBPA* had the best parameters among all assessed genes (AUC=0.96, Sensitivity=0.90 and specificity=0.97). *DILC* and *ATG5* were the most appropriate markers after *CEBPA* with AUC values of 0.82 and 0.80, respectively. Most notably, combination of all genes improved AUC, sensitivity and specificity parameters to 1, 0.97 and 0.99, respectively. Cumulatively, the current study provides evidence for participation of NF-κB-related lncRNAs in the pathoetiology of PD.

## Introduction

As the second most prevalent neurodegenerative disease, Parkinson’s disease (PD) has been shown to affect approximately 1% of the persons aged more than 65 years (1). From a neuropathological point of view, PD is associated with α-synuclein-comprising Lewy body and defects in dopaminergic neurons of substantia nigra which result in reduction of speed of voluntary movements (1). Although the main pathoetiology of PD is not clear, inflammation-related oxidative stress and cytokine-associated neurotoxic events have been shown to be involved in the stimulation of degradation of dopaminergic neurons (2, 3). Nuclear factor-κB (NF-κB) has been demonstrated to regulate activity of inflammatory intermediates in the course of inflammation. This transcription factors is expressed in microglia, neurons, and astrocytes in the central nervous system and contribute in the neurodegenerative process in PD (2). A previous study has shown significant increase in the percentage of dopaminergic neurons expressing NF-κB in their nuclei in PD patients compared with controls. Notably, there has been a possible association between the nuclear immunoreactivity for NF-κB in neurons of mesencephalon of these individuals and presence of oxidative stress in these neurons (4). Therapeutic intervention with NF-κB signaling has been suggested as a new strategy for management of inflammatory response triggered in the course of PD. In fact, agents that inhibit IKKβ or IKKγ have been shown to suppress neurodegeneration of dopaminergic neurons in animal models of PD (5). Thus, identification the regulatory mechanisms for modulation of NF-κB signaling in PD is an important issue for implementation of appropriate treatments for this disorder. Recent studies have revealed interactions between this signaling pathway and several of non-coding RNAs (6). These transcripts, particularly long non-coding RNAs (lncRNAs) have been suggested to participate in the pathophysiology of neuropsychiatric disorders, such as schizophrenia (7). In the current project, we compared expression levels of NF-κB-related lncRNAs and mRNAs, namely *CEBPA*, *ATG5*, *PACER*, *DILC*, *NKILA*, *ADINR*, *DICER1-AS1*, *HNF1A-AS1*, *CHAST* and *H19* in the circulation of PD patients *versus* healthy individuals to appraise their possible application as disease markers.

## Materials and Methods

### Patient and Controls

The current research was performed using the blood samples gathered from 50 cases of PD (14 females and 36 males) and 50 healthy individuals (15 females and 35 males). PD cases were recruited during January 2020-April 2021 from Farshchian hospital, Hamadan, Iran. PD cases were diagnosed based on criteria suggested by the International Parkinson and Movement Disorder Society (8). None of cases or controls had current or chronic infection, malignant conditions or any systemic disorders. Individuals recruited as controls had no personal or family history of any neuropsychiatric disorder. The study protocol was confirmed by ethical committee of Shahid Beheshti University of Medical Sciences. All PD patients and controls signed the informed consent forms.

### Expression Assays

A total of 3 mL of peripheral blood was gathered from PD cases and healthy controls in EDTA-blood collection tubes. Total RNA was extracted from these specimens using GeneAll extraction kit (Seoul, South Korea). Then, cDNA was produced from approximately 75 ng of RNA using BioFact™ kit (Seoul, South Korea). The Ampliqon real time PCR master mix (Denmark) was used for making reactions. Tests were executed in StepOnePlus™ RealTime PCR System (Applied Biosystems, Foster city, CA, USA). **Table 1** demonstrates primers sequences. PCR program consisted of a primary activation stage for 5 minutes at 94°C, and 40 cycles at 94°C for 15 seconds and 60°C for 45 seconds.

**Table 1:** Primer sequences.

Gene	Primer sequence		Product size (bp)
*CEBPA*	Forward	ACTTGGTGCGTCTAAGATGAGG	144
	Reverse	CATTGGAGCGGTGAGTTTGC	
*ATG5*	Forward	TTCGAGATGTGTGGTTTGGAC	134
	Reverse	CACTTTGTCAGTTACCAACGTCA	
*PACER*	Forward	TGGTCCTAAGCAGTTACCCTGTA	177
	Reverse	ACCAAAATAATCCACGCATCAGG	
*DILC*	Forward	GGAAAGGAGAGAAGAATGG	144
	Reverse	GTAAGATGTGGTTGTCGG	
*NKILA*	Forward	AACCACTATCATTTTATTTTCCATT	100
	Reverse	CAAAGCAATTCTCCTTTCCTA	
*ADINR*	Forward	TGGATGTGCTGTGATGAAGAGAAG	91
	Reverse	CCATAACACCTCCGCAGACAAATC	
*DICER1-AS1*	Forward	CCCAGCCTGCTTCCTGTTTTAAC	126
	Reverse	TTCTCTCCCATCTTCACCTTCTCC	
*HNF1A-AS1*	Forward	CCAGCCTGACCTCTCCATTCC	158
	Reverse	GCCGAACTGACATCACTGAACAC	
*CHAST*	Forward	GCAGAGGGTGCCAACTTGTA	109
	Reverse	TCTCAGGGAAATCAGATTGCGG	
*H19*	Forward	TGCTGCACTTTACAACCACTG	105
	Reverse	ATGGTGTCTTTGATGTTGGGC	
*B2M*	Forward	AGATGAGTATGCCTGCCGTG	104
	Reverse	CGGCATCTTCAAACCTCCA	

### Statistical Methods

Relative amounts of *CEBPA*, *ATG5*, *PACER*, *DILC*, *NKILA*, *ADINR*, *DICER1-AS1*, *HNF1A-AS1*, *CHAST* and *H19* were quantified in all samples relative to amounts of *B2M* transcripts. The Ln [Efficiency^ΔCT] formula was used for calculation of expression levels. Data was analyzed using R programming language and Rstan, ggplot 2 and non-parametric quantile regression packages. Mean values were compared between PD patients and healthy subjects using t-test. Spearman correlation coefficient was calculated to evaluate correlations between expressions of *CEBPA*, *ATG5*, *PACER*, *DILC*, *NKILA*, *ADINR*, *DICER1-AS1*, *HNF1A-AS1*, *CHAST* and *H19* genes. Receiver operating characteristic curves were plotted using and values for area under these curves (AUC) were measured.

## Results

**Table 2** shows the demographic data of PD patients and control subjects.

**Table 2:** Demographic and clinical profiles of PD patients and healthy controls [The Mini-Mental State Examination (MMSE), Unified Parkinson’s Disease Rating Scale (UPDRS)].

Parameters	Groups		Values
Sex (number)	Male		37
	Female		13
Age [Years, mean ± SD (range)]	Male		69.64 ± 10.59 (47–89)
	Female		66.46 ± 12.6 (38-85)
Duration [Years, mean ± SD (range)]	Male		3.18 ± 3.65 (1-12)
	Female		5.38 ± 9.76 (1-36)
MMSE [mean ± SD (range)]	Male		22.84 ± 3.032 (17-29)
	Female		23.08 ± 2.499 (19-26)
UPDRS [mean ± SD (range)]	Male		23.92 ± 7.418 (13-41)
	Female		26.31 ± 9.437 (16-42)
Hoehn & Yahr stage (number)	I	Male	8
		Female	3
	II	Male	18
		Female	5
	III	Male	11
		Female	5
Drug administration (number)	L-DOPA		46
	Bromocriptine, Amantadine, Quetiapine		4

**Figure 1:**
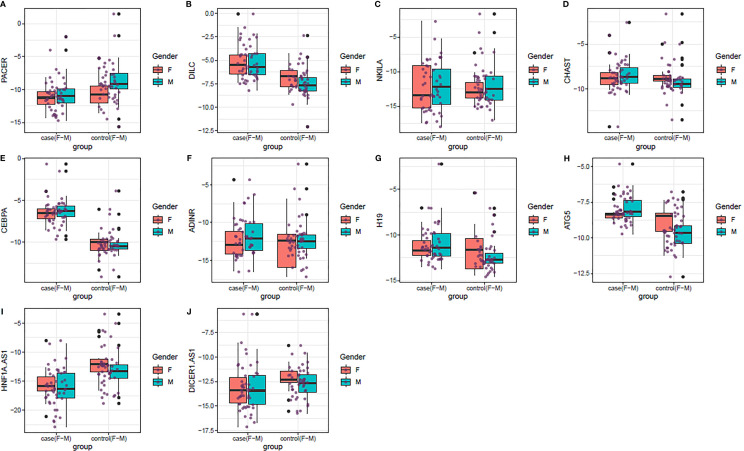
**(A–J)** Box-and-whisker plots showing the minimum values, the first quartiles, the medians, the third quartiles, and the maximum values of relative expressions of genes in PD cases and controls based on their gender (Red and blue plots show respective values among females and males, respectively).

**Figure 1** shows the minimum values, the first quartiles, the medians, the third quartiles, and the maximum values of relative expressions of genes in the formats of box-and-whisker plots.

Expression of *PACER* was lower in total PD patients compared with controls (Ratio of mean expressions (RME)=0.32, P value<0.001). This pattern was also evident among males (RME=0.25, P value<0.001). Expression of *DILC* was higher in total PD patients (RME=4.07, P value<0.001), and in both sex-based subgroups (RME=3.77, P value=0.01 and RME=4.25, P value<0.001, for females and males, respectively). Similarly, *CEBPA* was significantly over-expressed in total PD patients (RME=14.76, P value<0.001), and in both sex-based subgroups (RME=12.42, P value<0.001 and RME=15.80, P value<0.001, for females and males, respectively). ATG5 had a similar expression pattern (RME=2.6, P value=1E-08, RME=1.73, P value=0.03 and RME=3.09, P value=1E-07, for total cases, females and males, respectively). H19 was up-regulated in total cases and male cases compared with corresponding controls (RME=2.19, P value<0.001, RME=2.68, P value=0.01, respectively). Finally, *HNFA1-AS* was down-regulated in all comparisons (RME=0.10, P value=2E-06, RME=0.08, P value<0.001 and RME=0.12, P value<0.001, for total cases, females and males, respectively). Expression levels of other genes were not different between PD patients and controls (**Table 3**).

**Table 3:** Statistical parameters calculated for comparisons of genes expressions between PD patients and controls (SE, standard error, RME, ratio of mean expressions, CI, confidence interval).

Number of Samples		PACER					DILC					NKILA					CHAST					CEBPA				
Case/Control		SE	RME	P Value	95% CI		SE	RME	P Value	95% CI		SE	RME	P Value	95% CI		SE	RME	P Value	95% CI		SE	RME	P Value	95% CI	
**Total**	**50/50**	0.55	0.32	0.00	-2.72	-0.55	0.33	4.07	0.00	1.36	2.69	0.64	0.97	0.94	-1.32	1.22	0.35	1.32	0.26	-0.30	1.10	0.31	14.76	0.00	3.26	4.51
**F**	**14/15**	0.77	0.57	0.31	-2.39	0.78	0.65	3.77	0.01	0.55	3.28	1.26	1.20	0.84	-2.39	2.91	0.67	0.91	0.84	-1.55	1.27	0.53	12.42	0.00	2.55	4.72
**M**	**36/35**	0.69	0.25	0.00	-3.38	-0.65	0.39	4.25	0.00	1.31	2.87	0.76	0.88	0.81	-1.70	1.33	0.42	1.54	0.15	-0.22	1.46	0.39	15.80	0.00	3.21	4.76
		**ADINR**					**H19**					**ATG5**					**HNF1A-AS1**					**DICER1-AS1**				
**Number of Samples**		**SE**	**RME**	**P Value**	**95% CI**		**SE**	**RME**	**P Value**	**95% CI**		**SE**	**RME**	**P Value**	**95% CI**		**SE**	**RME**	**P Value**	**95% CI**		**SE**	**RME**	**P Value**	**95% CI**	
**Case/Control**																										
**Total**	**50/50**	0.52	1.31	0.45	-0.63	1.42	0.39	2.19	0.00	0.37	1.90	0.22	2.6	1E-08	0.94	1.82	0.65	0.10	2E-06	-4.56	-1.99	0.41	0.69	0.21	-1.343	0.30
**F**	**14/15**	1.11	1.89	0.42	-1.36	3.20	0.79	1.34	0.59	-1.20	2.05	0.35	1.73	0.03	0.07	1.51	1.14	0.08	0.00	-5.97	-1.29	0.75	0.50	0.20	-2.54	0.57
**M**	**36/35**	0.58	1.12	0.78	-0.99	1.31	0.44	2.68	0.01	0.56	2.30	0.27	3.09	1E-07	1.09	2.17	0.78	0.12	0.00	-4.66	-1.55	0.49	0.78	0.49	-1.34	0.65

**Figure 2:**
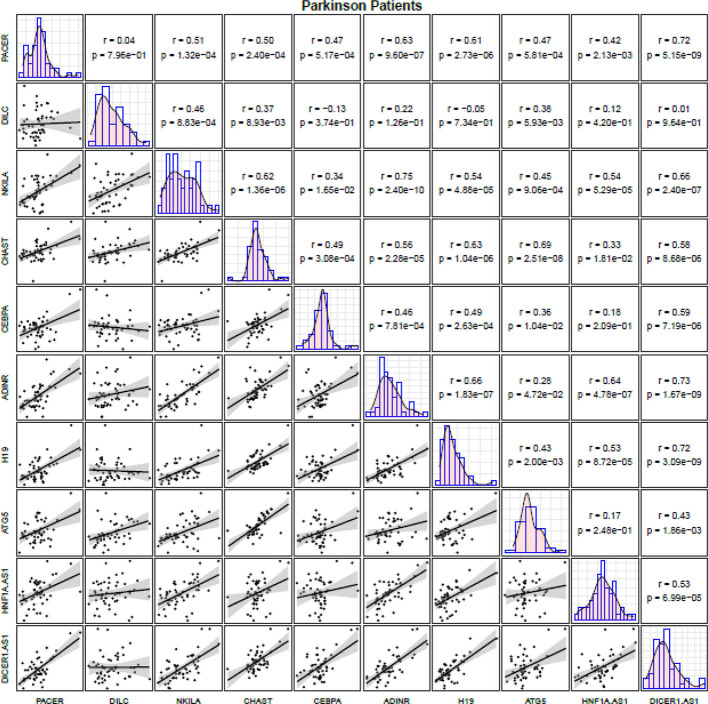
Correlation matrix for illustration of correlation between expression levels of NF-κB-related lncRNAs among PD patients (The distribution of expression levels of each lncRNA in PD patients is shown on the diagonal. Bivariate scatter plots are shown on the bottom of the diagonal. R and P values are shown on the top of the diagonal).

Among PD patients, expressions of NKILA and ADINR were robustly correlated with each other (r=0.75, P value=2.40E-10). In addition, expression levels of DICER1-AS were significantly correlated with those of ADINR, PACER and H19 in these patients (r=0.73, P value=1.76E-9; r=0.72, P value=5.15E-09 and r=0.72, P value=3.09E-09, respectively) (**Figure 2**).

**Figure 3:**
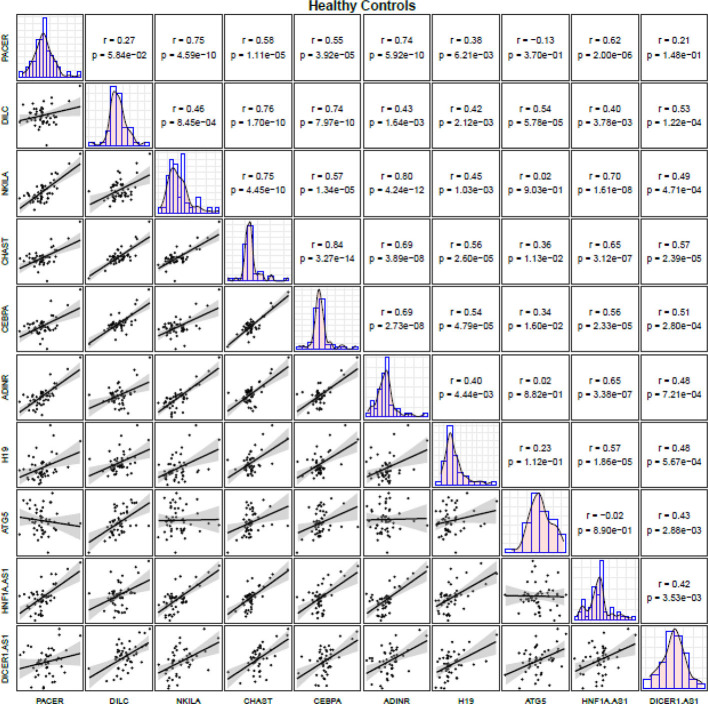
Correlation matrix for illustration of correlation between expression levels of NF-κB-related lncRNAs among controls (The distribution of expression levels of each lncRNA in controls is shown on the diagonal. Bivariate scatter plots are shown on the bottom of the diagonal. R and P values are shown on the top of the diagonal).

Correlation analyses among healthy controls revealed robust correlations between *CHAST* and *CEBPA* (r=0.84, P value=3.09E-09), *NKILA* and *ADINR* (r=0.80, P value=4.24E-12) as well as between *DILC* and *CHAST* (r=0.76, P value=1.70E-10). **Figure 3** illustrates correlation matrix for healthy controls.

**Figure 4:**
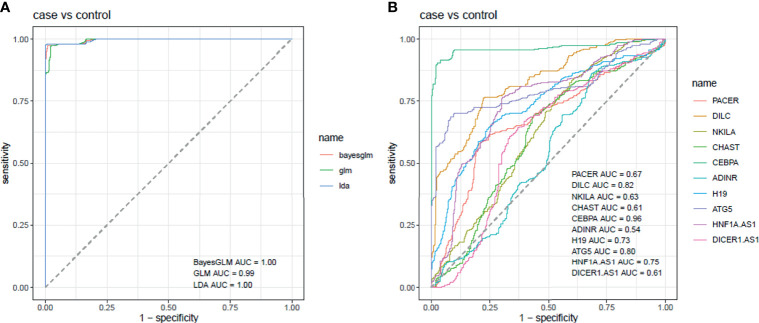
ROC curves for assessment of diagnostic power of these genes using three distinctive models **(A)**. Assessment of diagnostic values of NF-κB-related genes in PD using Bayesian GLM **(B)**.

Then, we depicted ROC curves for assessment of diagnostic power of these genes using three distinctive models with Bayesian GLM showing the best values (**Figure 4A**). Subsequently, this model was used for plotting ROC curves for all genes (**Figure 4B**).

*CEBPA* had the best parameters among all assessed genes (AUC=0.96, Sensitivity=0.90 and specificity=0.97). *DILC* and *ATG5* were the most appropriate markers after *CEBPA* with AUC values of 0.82 and 0.80, respectively. Most notably, combination of all genes improved AUC, sensitivity and specificity values to 1, 0.97 and 0.99, respectively (**Table 4**).

**Table 4:** Statistical parameters of ROC curve analyses.

PACER			DILC			NKILA			CHAST			CEBPA			ADINR			H19			ATG5			HNF1A-AS1			DICER1-AS1			All Markers		
AUC	Sensitivity	Specificity	AUC	Sensitivity	Specificity	AUC	Sensitivity	Specificity	AUC	Sensitivity	Specificity	AUC	Sensitivity	Specificity	AUC	Sensitivity	Specificity	AUC	Sensitivity	Specificity	AUC	Sensitivity	Specificity	AUC	Sensitivity	Specificity	AUC	Sensitivity	Specificity	AUC	Sensitivity	Specificity
0.66	0.58	0.80	0.82	0.76	0.78	0.63	0.83	0.40	0.61	0.70	0.57	0.96	0.90	0.97	0.53	0.86	0.31	0.73	0.64	0.76	0.80	0.70	0.90	0.75	0.76	0.70	0.61	0.64	0.63	1	0.97	0.99

## Discussion

NF-κB comprise a group of transcription factors that through regulation of inflammation and apoptosis contribute in the programming of systemic ageing in the central nervous system and pathobiology of the neurodegenerative disease PD (9). NF-κB-associated genes partake in the regulation of the cellular levels of reactive oxygen species in the cell (10, 11). Moreover, through regulating autophagic processes, NF-κB acts as an important modulator of clearance of protein aggregates (12). Notably, NF-κB has been shown to be activated in microglia upon their exposure to lipopolysaccharide. Activation of this nuclear factor enhances expression of proinflammatory genes as well as proteolytic enzymes (13). Based on the structure of NF-κB dimers, this pathway might exert either protective or harmful effects. To be more precise, p50/RelA and c-Rel-containing dimers have pro-apoptotic and neuroprotective effects, respectively (14, 15). In the current investigation, we compared expression levels of a number of NF-κB-related lncRNAs and mRNAs in the circulation of PD patients *versus* healthy controls. Expression of *PACER* was lower in total PD patients compared with controls. This pattern was also evident among males. Moreover, *HNFA1-AS* was down-regulated in all comparisons. We have recently reported down-regulation of *PACER*, while up-regulation of *HNFA1-AS* in patients with schizophrenia compared with normal individuals (7), indicating distinctive roles of these lncRNAs in these two neuropsychiatric conditions. *PACER* is an lncRNA whose expression is induced by cyclooxygenase-2. This lncRNA has a functional association with p50, a suppressive subunit of NF-κB, and obstructs it from the promoter of the cyclooxygenase-2 gene, thus enhancing the interplay with activating NF-κB p65/p50 dimers (16). *HNF1A-AS1* is a natural antisense RNA for *HNF1A* (17) whose expression is increased by HNF1A (18). NF-κB *via* affecting expression of TNF-α decreases levels of HNF1A (19), thus it is expected to decrease expression of *HNF1A-AS1*. Therefore, the reduced levels of *HNF1A-AS1* in PD patients might be explained by higher activity of NF-κB signaling in these patients.

Expression of *DILC* was higher in total PD patients, and in both sex-based subgroups. This lncRNA has been shown to inhibit the autocrine IL-6/STAT3 axis (20). STAT3 has a possible impact in the pathogenesis of PD, since the PD gene, *DJ-1* has been shown to regulate astrogliosis *via* this factor (21). Moreover, STAT3 can trigger production of neurotoxic proteins by microglia (22). The observed up-regulation of *DILC* in PD patients can be a compensatory response to attenuate the harmful effects of IL-6/STAT3 axis in these patients.

Similarly, *CEBPA* was significantly over-expressed in total PD patients, and in both sex-based subgroups. *CEBPA* has been among genes of interest for PD recognized by “guilt-by-association” with the known PD-associated genes (23). *CEBPA* has also been reported to interact with the promoter region of leptin coding gene and regulate its expression. Leptin can be easily transported to the brain and interact with its receptors in neurons to influence neurodevelopment (24).

*ATG5* had a similar high expression pattern in both sex-based subgroups of PD patients. Over-expression of ATG5 has been shown to protect dopaminergic neurons in an animal model of PD (25). Thus, upregulation of this gene in PD patients might be a compensatory response to attenuate neuron loss in these patients.

Finally, *H19* was up-regulated in total cases and male cases compared with corresponding controls. This lncRNA reduces dopaminergic neuron loss in PD through modulation of Wnt/β-catenin signaling (26). Moreover, it decreases apoptosis in MPTP-associated PD *via* modulation of miR-585-3p/PIK3R3 axis (27).

Among PD patients, expressions of *NKILA* and *ADINR* were robustly correlated with each other. In addition, expression levels of *DICER1-AS* were significantly correlated with those of *ADINR*, *PACER* and *H19* in these patients. Correlation analyses among healthy controls revealed robust correlations between *CHAST* and *CEBPA*, *NKILA* and *ADINR* as well as between *DILC* and *CHAST*. Thus, the pattern and robustness of correlations were affected by the presence of PD, except for *NKILA* and *ADINR* genes which were robustly correlated in both groups of study participants.

*CEBPA* had the best parameters among all assessed genes (AUC=0.96, Sensitivity=0.90 and specificity=0.97). *DILC* and *ATG5* were the most appropriate markers after *CEBPA* with AUC values of 0.82 and 0.80, respectively. Most notably, combination of all genes improved AUC, sensitivity and specificity values to 1, 0.97 and 0.99, respectively. Therefore, this study provides clues for design of a panel of genes for diagnosis of PD or follow-up of patients. Assessment of expression profile of these genes during different stages of development of PD as well as in drug-naïve patients would help in identification of biomarker role of these genes.

Cumulatively, the current study provides evidence for participation of NF-κB-related lncRNAs in the pathoetiology of PD. Modulation of immune responses and apoptotic pathways are the most probable mechanisms of participation of these lncRNAs in the pathoetiology of PD. We recommend conduction of functional studies for appraisal of the mechanisms of involvement of these genes in the pathogenesis of PD.

Our study has some limitations. First, we did not include a group of drug-naïve patients to apprise expression of these genes in them. Second, we did not perform *in vitro* or *ex vivo* studies to unravel the mechanism of involvement of these genes in the pathogenesis of PD.

Future studies are needed to assess expression levels of NF-κB-related lncRNAs in larger cohorts of PD patients to verify their diagnostic impact. Moreover, the effect of modification of their expression on the course of PD should be assessed in animal models of PD in roder to find novel therapeutic options for this disorder.

## Data Availability Statement

The raw data supporting the conclusions of this article will be made available by the authors, without undue reservation.

## Ethics Statement

The studies involving human participants were reviewed and approved by Shahid Beheshti University of Medical Sciences IR.SBMU.MSP.REC.1400.152. The patients/participants provided their written informed consent to participate in this study.

## Author Contributions

MT and SG-F wrote the draft and revised it. MT and AS designed and supervised the study. MG and AA performed the experiment. MM collected the samples and data. All authors contributed to the article and approved the submitted version.

## Conflict of Interest

The authors declare that the research was conducted in the absence of any commercial or financial relationships that could be construed as a potential conflict of interest.

## Publisher’s Note

All claims expressed in this article are solely those of the authors and do not necessarily represent those of their affiliated organizations, or those of the publisher, the editors and the reviewers. Any product that may be evaluated in this article, or claim that may be made by its manufacturer, is not guaranteed or endorsed by the publisher.
